# The Role of Resveratrol in Mammalian Reproduction

**DOI:** 10.3390/molecules25194554

**Published:** 2020-10-05

**Authors:** Rolando Pasquariello, Nicole Verdile, Tiziana A. L. Brevini, Fulvio Gandolfi, Cristiano Boiti, Massimo Zerani, Margherita Maranesi

**Affiliations:** 1 Department of Agricultural and Environmental Sciences, University of Milan, 20133 Milano, Italy; rolando.pasquariello@unimi.it (R.P.); nicole.verdile@unimi.it (N.V.); 2 Department of Health, Animal Science and Food Safety, University of Milan, 20133 Milano, Italy; tiziana.brevini@unimi.it; 3 Department of Veterinary Medicine, University of Perugia, 06126 Perugia, Italy; boiti.cristiano@gmail.com (C.B.); margherita.maranesi@unipg.it (M.M.)

**Keywords:** polyphenols, reproduction, phytoestrogens, ovary function, sirtuin, testis function, spermatozoa, cryopreservation, sperm quality, oocyte quality

## Abstract

Resveratrol is one of the most investigated natural polyphenolic compounds and is contained in more than 70 types of plants and in red wine. The widespread interest in this polyphenol derives from its antioxidant, anti-inflammatory and anti-aging properties. Several studies have established that resveratrol regulates animal reproduction. However, the mechanisms of action and the potential therapeutic effects are still unclear. This review aims to clarify the role of resveratrol in male and female reproductive functions, with a focus on animals of veterinary interest. In females, resveratrol has been considered as a phytoestrogen due to its capacity to modulate ovarian function and steroidogenesis via sirtuins, SIRT1 in particular. Resveratrol has also been used to enhance aged oocyte quality and as a gametes cryo-protectant with mainly antioxidant and anti-apoptotic effects. In males, resveratrol enhances testes function and spermatogenesis through activation of the AMPK pathway. Furthermore, resveratrol has been supplemented to semen extenders, improving the preservation of sperm quality. In conclusion, resveratrol has potentially beneficial effects for ameliorating ovarian and testes function.

## 1. Introduction

**Figure 1:**
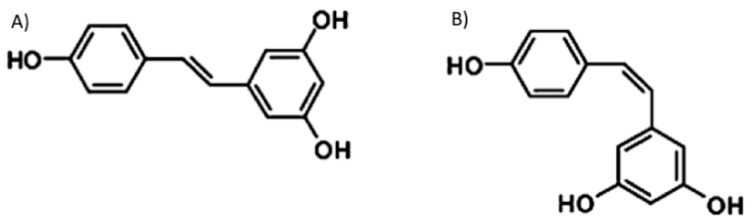
Chemical structures of (**A**) trans- and (**B**) cis- resveratrol.

Resveratrol (3,5,4′-trihydroxystilbene) is a polyphenol that belongs to dietary stilbenes, a class of natural compounds that display significant biological activities of medicinal interest. This compound is one of the best known and most investigated polyphenols found in nature, is produced by more than 70 different types of plants, and is contained in red wine and in several botanical extracts [1]. The importance of resveratrol in medicine has been known since the 1940s; it was, in fact, initially extracted from plant roots and successfully used in traditional Japanese and Chinese medicine [2,3]. Resveratrol derives from phenylalanine through the activation of the enzyme stilbene synthase and exists in two isomeric forms, trans- and cis- resveratrol [4] (Figure 1). Trans-resveratrol is the most common form in plants and the most widely investigated; therefore, in the present review, we will mainly deal with this isoform. Plants synthesize resveratrol and other stilbenes in response to stressful conditions including ultraviolet irradiation, extreme temperatures, mechanical damages, and the activity of microorganisms such as fungi and bacteria [5].

Therefore, this phenolic compound was initially characterized as a phytoalexin for its fungicidal role [6], and only later was it appreciated as a nutrient supplement, for several beneficial effects in the prevention and improvement of various diseases highlighted over the years [7]. Resveratrol is often associated with the “French paradox”, a term coined in the early 90s, based on epidemiological data from French people having a low incidence of coronary pathologies and generally a long life expectancy likely due to moderate consumption of red wine despite a diet characterized by high saturated fat intake [8]. Since its discovery, resveratrol has been considered to be effective in improving health and preventing chronic disorders, like ischemic and atherosclerotic injuries, neurodegenerative diseases, and metabolic diseases (e.g., diabetes) [7], thanks to its anti-inflammatory and antioxidant effects [9]. The antioxidative effects of resveratrol are related to the high redox property of phenolic hydroxyl groups, which act as free radical scavengers. Resveratrol activates many antioxidant enzymes such as catalase and superoxide dismutase [10].

Excellent and exhaustive reviews on the role of resveratrol on pregnancy [11], on the ovarian and endometrial function [12,13,14], and on the androgenic production of Leydig cells [15] have been published. This review aims to summarize the available data on the possible role of resveratrol on reproduction, with a particular focus on animals of veterinary interest. In particular, it will clarify the effect of resveratrol on both female and male reproduction and discuss the mechanisms of action.

## 2. Resveratrol Effects on Females

### 2.1. Resveratrol as a Phytoestrogen

Despite numerous studies, it is still debated whether resveratrol can be used alone or in combination with other estrogenic substances to regulate the reproductive function of animals or be used in estrogen replacement therapy of women [13,14]. With this aim, many authors have investigated the mechanism of action of resveratrol in different target organs in vitro [16,17] and in vivo [18].

Because resveratrol has a chemical structure similar to that of some estrogens, such as diethylstilbestrol (DES), it is considered a natural phytoestrogen [16,17]. The cardioprotective activity [19] and estrogen-dependent cancer protection role of estrogens [20,21] are well-documented. Numerous studies have described the same estrogenic role of resveratrol in these disorders [22,23,24,25,26].

Estrogens, including phytoestrogens, act via the estrogen receptors (ERs), members of the nuclear receptor superfamily. Many chemicals of plant origin such as genistein, coumestrol, and resveratrol contain one or two six-carbon rings with hydroxyl substituents that can mimic estradiol; therefore, such a phytochemical compound is an agonist for the two receptor subtypes, ER alpha and ER beta [27]. The transcription is activated at the same nuclear level for both estrogen and phytochemical compounds, in the peculiar tract of the estrogen response element [16].

Since 1997, Gehm et al. [17], using different assays, demonstrated that resveratrol is a phytoestrogen that acts via binding to ERs and has different agonist possibilities dependent on the system in which it is assayed. Stahl et al. [28] described positive estrogenic effects of different phytoestrogenic compounds such as genistein, coumestrol, and zearalenone on estrogen-dependent pituitary tumor cells. By Western blot, the authors found only the ER alpha expression in this cell line, supposing that these phytoestrogen responses were mediated by this isoform [28]. Conversely, resveratrol did not bind and had no subsequent growth activity in the same cell lines, despite it inducing prolactin secretion and mRNA up-regulation, and both effects were blocked by estrogen antagonists. In these cell lines, resveratrol probably acts independently of the binding to ER alpha, but, in any case, it shows an effect comparable to that of other tested phytoestrogens [28].

In 2002, Henry et al. [18] examined the effect of resveratrol administered to female rats in vivo. Although resveratrol did not show high affinity for ERs, it was still able to determine effects on hypothalamic–pituitary–gonadal axis regulatory genes, affecting the estrous cycles and inducing gonad hypertrophy in intact animals. Instead, resveratrol did not replace the effect induced by 17-beta estradiol in rat gonadectomized females [18].

In Chinese Hamster Ovary cells (CHO-K1) it was found that resveratrol binds the two ER receptors (ER alpha and beta) with a similar affinity, but with an affinity approximately 7,000 times lower than estradiol [16]. This is in contrast with findings obtained for other phytoestrogens, which bind the beta form of ERs with higher affinity than the alpha form [29]. Interestingly, also DES, which has a structural analogy with resveratrol [17], shows a greater affinity for the alpha form of the ERs [29].

Although there are conflicting data regarding resveratrol as an agonist of ERs, the findings testify to a potential role of this compound in enhancing the estrogenic effects of hormones and therefore as a modulator of the reproductive function.

### 2.2. Mechanisms of Action: Sirtuins

**Figure 2:**
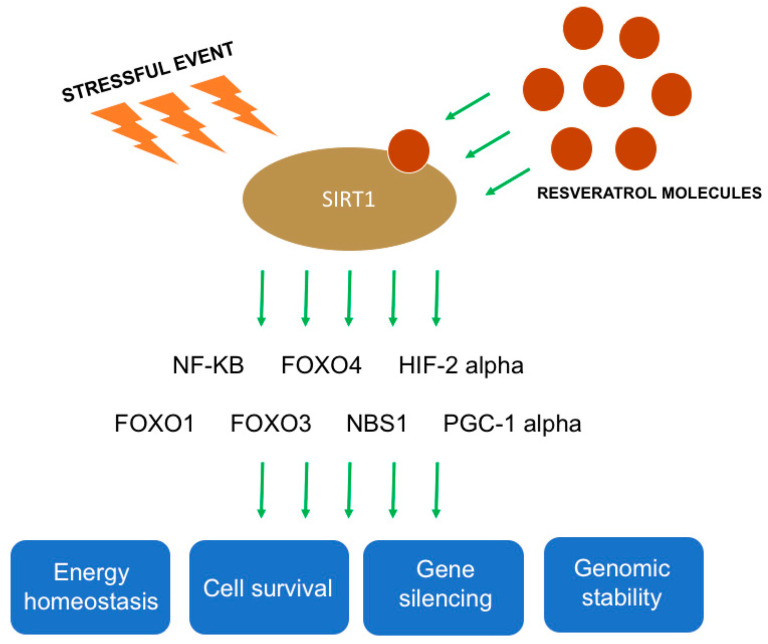
Mechanisms of action of resveratrol on silent information regulator 2 type 1 (SIRT1). Following a stressful event, resveratrol activates SIRT1 that binds different genes such as NF-kB, FOXO4, HIF 2 alpha, FOXO1, FOXO3, NBS1, and PGC-1 alpha. The activation of these genes is correlated with regulation of energy homeostasis, cell survival, gene silencing, and genomic stability.

Growing evidences indicate the role of resveratrol in ovarian function and steroidogenesis modulation mediated by sirtuins [12,14] (Figure 2).

Sirtuins are proteins of the nicotinamide adeninedinucleotide-dependent deacetylases family (or silent information regulator 2 family—SIRT family), which are well-known for their role in many cellular processes [30] such as apoptosis [31], cell reprogramming [32], and DNA repair [33]. Sirtuins are also involved in cancer progression [34], ovarian aging [35,36], redox homeostasis [37], and glucose and lipid metabolism [38]. Since modification of the NAD+/NADH ratio controls the activity of SIRTs, all members of this family have a pivotal role in sensing the oxidative stress and energetic condition of the cell [39].

To date, seven members of the sirtuin family have been identified in mammals (SIRT1-7), each member playing a role in ovarian function (for an extensive review, see [39,40]). In fact, damage impairing SIRT’s activity leads to fertility deficits [39,41,42].

Resveratrol is the most potent natural ligand of silent information regulator 2 type 1 (SIRT1). After a stressful event, SIRT1 is activated and binds different molecular targets, including nuclear factor kappa-light-chain-enhancer of activated B cells (NF-kB), tumor protein p53, forkhead box (FOX) -O1 (FOXO1), -O3 (FOXO3), and -O4 (FOXO4), peroxisome proliferator-activated receptor-gamma coactivator (PGC-1 alpha), liver X receptor, nibrin (NBS1), and hypoxia-inducible factor 2 alpha (HIF-2 alpha) (Figure 2) [43,44,45]. By activating these molecules, via SIRT1, resveratrol has a pivotal role in regulating energy homeostasis, gene silencing, genomic stability, and cell survival (Figure 2) [43].

Resveratrol may also protect against ovarian aging through SIRT1-related cellular mechanisms, exerting an anti-oxidative effect that guards oocytes from age-dependent deficits [39].

In rat granulosa cells, resveratrol induced a transcript up-regulation of SIRT1, LH receptor, StAR, and P450 aromatase, while mRNA levels of FSH receptor remained unchanged [46], thus suggesting that resveratrol and SIRT1 can modulate ovarian functions via folliculogenesis-related molecules and gonadotropin receptor activation.

In swine granulosa cells, resveratrol increased SIRT1 mRNA and protein level in a dose-dependent fashion, accelerating cell apoptotic rate and follicular atresia [47]. Resveratrol supplemented in cultured porcine ovarian granulosa cells determined SIRT1 protein increase and apoptosis, promoting testosterone and estrogen release, while inhibiting cell proliferation [42].

When supplemented to in vitro maturation (IVM) medium, polydatin, a glycosidic form of resveratrol, improved embryo development, increasing SIRT1 protein and decreasing reactive oxygen species (ROS) [48]. In the same work, embryo protein levels of nuclear factor NF-kB and cyclooxygenase (COX2) were significantly lower when polydatin was added to the culture medium [48]. Since NFkB and especially COX2 play a pivotal role in inflammation, the authors supposed that this resveratrol analogue (polydatin) might have a beneficial effect on embryo development by decreasing their expression, thereby reducing any inflammatory processes in progress [48]. Using immunofluorescence and Western blot techniques, Wang et al. [49] evidenced the presence of SIRT1 in bovine granulosa cells, cumulus cells, oocytes, and blastocysts. Moreover, resveratrol increased SIRT1 mRNA and protein levels in cumulus cells [49]. These authors suggested that the beneficial effects of resveratrol on oocyte maturation and embryonic development after in vitro fertilization might be SIRT1-mediated [49].

Thus, there is enough evidence that resveratrol may have a positive effect on the reproductive function via sirtuins and specifically via SIRT1 even if the possibility that resveratrol acts through other pathways cannot be excluded.

### 2.3. Effects of Resveratrol on Oocyte and Embryo

Resveratrol interferes with the endocrine and paracrine communications taking place between the cumulus oophorous and the oocyte. In domestic species, it is well documented that resveratrol: enhances maturation and quality of aged oocytes [50], is an effective cryo-protectant with antioxidant and anti-apoptotic effects [51], and increases the embryo developmental competence to the blastocyst stage [52].

#### 2.3.1. Oocyte Maturation

Phytomelatonin is a well-known product used in phytomedicine for its antioxidant properties [53]. In this context, Lee et al. [54] investigated the synergistic properties of melatonin and resveratrol to ameliorate porcine IVM of oocytes. These authors found that the association of the two compounds in the medium of cumulus-oocyte complexes undergoing IVM supported a synergistic increase in oocyte nuclear maturation and total cell numbers of parthenogenetic activated blastocysts, and improved the development of somatic cell nuclear transfer embryos [54]. In another study, in cattle, supplementation of IVM medium with different antioxidants, including resveratrol, was correlated with decreased ROS levels and increased GSH levels in the oocytes [55]. Similarly, supplementation with 20 µM of resveratrol improved the quality of bovine oocyte, which matured in vitro by ameliorating mitochondrial quantity and quality, ATP content, and fertilization rate, via SIRT1 up-regulation [56].

#### 2.3.2. Oocyte Cryopreservation

Cat immature oocytes contain a large-sized germinal vesicle with decondensed chromatin that is highly susceptible to cryo-damage [51]. The histone deacetylase enhancer activity of resveratrol prevents cryopreservation damage during oocytes vitrification [57]. Comizzoli et al. examined the use of resveratrol as an adjuvant in cryopreservation, revealing that transient epigenetic modifications associated with chromatin compaction of germinal vesicle induced by resveratrol were fully reversible and beneficial to oocyte survival during vitrification. Resveratrol treatment in ovaries stored for 48 h at 4 °C can reverse the negative effect of oxidative stress in oocytes, with positive effects on embryo development [58]. In fact, resveratrol increased the glutathione (GSH) levels and reduced those of ROS in oocytes; in addition, it ameliorated blastocyst rate formation and cell number in the developed blastocysts [58].

Beneficial effects, such as positive modulation of the apoptotic process and improvement of porcine oocyte resistance, were obtained by Giaretta et al. [59] at the same dosage of resveratrol supplementation used by Lee et al. [52] in different phases of IVM and vitrification/warming procedure. Using the same resveratrol supplementation concentration (2.0 µM), Santos et al. [60] demonstrated a beneficial impact of resveratrol on the developmental competence of vitrified oocytes, only when added to the IVM medium, but not when resveratrol was added as a pre-treatment of the vitrification process.

Since during cryopreservation, functional aberrations in oocytes may intervene due to lipid content variation and formation of ROS, Sprícigo et al. [61] assessed the effect of L-carnitine and/or resveratrol addition to maturation medium before calf oocyte vitrification. L-Carnitine is known both for its modulating activity on lipid metabolism and for its antioxidant action [61]. L-Carnitine and resveratrol supplementation before vitrification decreased spindle damage, while resveratrol addition modulated apoptosis [61]. The addition of L-carnitine or resveratrol before vitrification positively affected the expression of genes of vitrified/warmed oocytes [61].

#### 2.3.3. Embryo Development

Lee et al. [52] examined different resveratrol dose effects on pig embryos obtained by parthenogenesis and/or IVF. The optimal dosage was found at 0.5 µM resveratrol, where (1) a higher percentage of parthenogenetic embryos reached the blastocyst stage at day 7 with a higher total blastocyst cell number; (2) resveratrol incubation negatively affected the expression levels of apoptosis-related genes in parthenogenetic blastocysts [52]. A lower expression of BCL2 and caspase-3 was observed, suggesting a positive effect for porcine embryos [52]. Similar conclusions, but with different dosages, were found by Kwak et al. [62]. The favorable effects were reached at 2.0 µM of resveratrol supplementation during in vitro maturation (IVM), improving the developmental potential of PA and IVF porcine embryos by increasing the intracellular GSH level, decreasing ROS level, and down-regulating apoptosis gene expression during oocyte maturation [62]. In cows, a moderate amount of resveratrol supplemented to the culture medium (0.5 µM) achieved positive effects on the embryo as suggested by the higher development and hatching rates recorded after 48 h post-warming culture [63]. Moreover, in this species, resveratrol supplemented to the in vitro cultured (IVC) medium and/or vitrification solution (VS), at 0.5 µM concentration to protect embryos from the negative effect of cryopreservation, partially restored their quality [64]. In fact, resveratrol addition to IVC medium partially compensated for the gene expression increase for FOXO3 and patatin-like phospholipase domain containing 2 (PNPLA2), but not for BCL2-like 1 and BCL2-associated X, apoptosis regulator (BAX), restoring GSH content in bovine embryos [64].

#### 2.3.4. Aged Oocyte

Maternal aging often impairs the quality of oocytes and embryos and affects, amongst others, mitochondrial function and numbers, and spindle assembly [65,66,67]. These alterations have been related to oxidative stress in human and mouse [65,66,67]. However, similar results have been found in cattle as well. Sugiyama et al. [50] collected oocytes and granulosa cell complexes from early antral follicles of aged cows (>10 age years) and examined the effects of resveratrol on mitochondrial generation, degradation, and quality in oocytes grown in vitro [50]. Interestingly, resveratrol affected both oocytes and granulosa cells, improving the quality of growing oocytes, through up-regulation of mitochondrial biogenesis and degradation of growing oocytes and by modulating genes in granulose cells whose expression levels are associated to the developmental competence of oocytes and embryos [50]. In the following study, it clearly appears that resveratrol ameliorates the quality of oocytes obtained from aged females. The positive effect of resveratrol on mitochondrial function has been proved in experiments performed on oocytes aged in vitro as well. These oocytes are generally obtained using time-dependent deterioration in quality [68] and, thereby, are different from the oocytes obtained from aged females. However, the two different sources of oocytes have similar alterations in the mitochondria, which determine loss of quality. In 2015, Ma et al. [69] reported that SIRT1 expression was notably reduced in pig oocytes that were aged in vitro. Resveratrol treatment during pig oocyte maturation reduced (probably via SIRT1) these defects [69]. In the following study, while SIRT1 impaired mitochondria number and function in the oocytes, the supplementation of IVM medium with resveratrol increased mitochondrial in the developing oocytes, thereby improving their own competence [69]. Overall, all these studies reported that the effect of resveratrol varied in a dose-dependent way and could be species-related. Moreover, here we clarify that resveratrol has favorable effects on mitochondria since it improves their function.

## 3. Resveratrol Effects on Males

### 3.1. Impact of Resveratrol on Male Reproductive Function and Spermatogenesis

Estrogens were identified in testes, where they play a paracrine regulatory function [70,71,72], suggesting a possible role for resveratrol, given its structural similarity to estradiol, as previously reported in this review. Several studies reported that resveratrol modulates the estrogen-response system, acting as a regulator of male reproductive function [73]. However, the role of resveratrol in male reproductive function is not clearly established yet, although considerable work has been done. Some studies indicate that resveratrol arguably improves sperm quality in humans [74,75] and domestic animals [76,77,78,79,80]. This seems to be possible thanks to its capacity to pass through the blood–testis barrier, imparting its protective effects in the testis [81]. Resveratrol administration was shown to: (1) decrease germ cell apoptosis [82,83], (2) trigger penile erection [82,83], (3) enhance serum testosterone concentration [82,83], and (4) improve sperm quality and epididymal sperm number [84]. These different actions of resveratrol on the male reproductive system resulted from a direct stimulation of the hypothalamic–pituitary–gonadal axis, with no adverse effects on testes [73]. Resveratrol administration in vivo was used to treat infertility. In men affected by dyszoospermia, resveratrol promoted spermatogenesis by ameliorating the effect induced by 2,5-hexanedione [73]. In this study, it was also established that the expression of c-kit, a specific marker protein of spermatogenic cell membranes, was regulated by resveratrol [73]. Resveratrol has been extensively used during cancer therapy since its positive impact in preserving male reproductive function has been demonstrated. In this scenario, resveratrol administration preserved the metabolic pathways involved in erectile function and provided functional protection of prostatic cancer patients undergoing radiotherapy [85]. Another recent study on the use of resveratrol during cancer therapy determined that the administration of resveratrol during treatment with paclitaxel, diminished DNA fragmentation of rabbit epididymal spermatozoa after cryopreservation [86]. All these results show that resveratrol not only modulates the male reproductive function, but is capable of exerting a direct and protective effect on spermatogenesis. Similar results have been observed in mice affected by cryptorchidism, whereby resveratrol was capable of preserving spermatogenesis after a daily dose treatment [79]. According to this study, the number of primary spermatocytes was higher in the histological section of treated cryptorchid males than in not-treated ones [79]. This effect was also found using resveratrol together with other antioxidant agents. Administration of resveratrol, alpha lipoic acid, and coenzyme Q10 was indeed correlated with a protective effect on radiation-induced spermatogenesis injury [87]. The results of this study demonstrated that resveratrol can act with other antioxidant molecules to enhance sperm maturation [87]. On the contrary, in the same study, no effect on the protection of Leydig cells as a source of testosterone was observed [87].

The positive effects of resveratrol have also been shown in metabolic disorders such as diabetes. Abdeli et al. [88] demonstrated that resveratrol ameliorated Type 1 diabetes mellitus-induced abnormal sperm formation, oxidative stress, and DNA damage and had some effects on PARP signaling pathway in the rat testis [88].

Despite considerable data on the effects of resveratrol, the mechanisms underlying this phenomenon are still unclear. According to several studies, resveratrol directly acts on the expression of sirtuin-1 [43,46,89]. According to Seneret al. [85], resveratrol increased the expression of sirtuin-1, neuronal nitric oxide synthase (nNOS), and endothelial NOS (eNOS) protein expressions of oncological patients treated using resveratrol during radiotherapy [85]. These findings indicate that resveratrol activates sirtuin-1 with subsequent activation of eNOS, leading to enhanced cyclic guanosine monophosphate synthesis via the nitric oxide/cyclic guanosine monophosphate pathway [90]. The activation of this pathway leads to a decreased rate of apoptosis [83] and stimulates germ cell differentiation [82,83,87].

Finally, the positive effect of resveratrol on male reproductive function has led to the study of its analogues that, on the contrary, exerts an inhibitory action on reproductive function. Svechnikow et al. [91] observed an inhibitory effect of resveratrol analogues on steroidogenesis in Leydig cells of rats, indicating novel mechanisms of action. The results of this study may be useful for developing potential therapies as a male contraceptive agent, where suppression of androgen action is needed [91].

### 3.2. Use of Resveratrol in Sperm Cryopreservation

Cryopreservation of sperm is commonly used for the management and long-term preservation of male fertility in humans and domestic animals [92,93]. However, freeze-thawing processes induce oxidative stress in mammalian spermatozoa because of the production of a large amount of ROS due to high concentration of poly-unsaturated fatty acids located on sperm membranes [81,94,95]. ROS negatively impact sperm quality and motility since they damage cellular proteins, DNA and plasma membrane lipids, with subsequent reduction of capacity of the spermatozoa to fertilize the oocyte [96].

In the ejaculate, the equilibrium of ROS level can be maintained because the seminal plasma contains antioxidant molecules, membrane stabilizers, and sugars [97,98], among others. However, during the procedure of dilution and cooling, the semen is markedly exposed to oxidative stress since spermatozoa do not have adequate reserves of natural antioxidants functioning to reduce the negative impact of ROS, which induces lipid peroxidation (LPO) during the preparation of sperm for cryopreservation [99,100], inducing sperm mitochondrial dysfunction that occurs because of temperature changes, ice formation, and osmotic stress [101]. Furthermore, ROS levels in the sperm significantly increased during the cryopreservation process [102].

The supplementation of antioxidants to the extenders has led to an enhancement in values for the post-thaw sperm quality variables in several species including bull [103], stallion [104], red deer stag [105], dog [106], ram [107], buck goat [108,109], and boar [110,111] semen.

In this scenario, resveratrol has been extensively used as a suitable antioxidant supplement to semen extenders in human, mouse, ram, bull, buffalo, and boar semen [75,86,112,113,114,115,116]. In particular, in vivo and in vitro studies indicated that resveratrol improves sperm quality during the cryopreservation process [74,87], thanks to its protective function against lipid peroxidation (LPO) and DNA damage caused by ROS [75,117].

In humans, resveratrol has been reported to minimize post-thawing DNA damage to spermatozoa [114]. Similarly, in cattle, it has been observed that supplementation of resveratrol in semen extenders improved post-thaw bull sperm quality, in terms of sperm motility, mitochondrial activity, and DNA integrity [114]. The ability of resveratrol to act as an antioxidant was also proved using induced oxidative stress in vitro, where it was reported that mouse [113], cattle [118], buffalo [116] and human spermatozoa [75] can be protected by resveratrol. Furthermore, in frozen-thawed ram sperm, the addition of resveratrol to the tris-egg yolk-glycerol extender was shown to reduce sperm mitochondrial membrane potential [119].There are many other studies that demonstrated how resveratrol may act as an antioxidant. All these studies lead to common conclusions that can be summarized in the ability of resveratrol to: (1) reduce ROS production in the mitochondria; (2) scavenge superoxide radicals, including superoxide anion, hydroxyl radical, and metal-induced radicals; (3) inhibit lipid peroxidation; and (4) regulate the expression of antioxidant cofactors and enzymes [120,121,122]. However, even if there is a clear positive effect on sperm quality, there are no data that indicate that resveratrol may improve motility of freeze-thawed spermatozoa. Moreover, Falchi et al. [123] did not find any antioxidant effect of resveratrol on the post-thawed buck semen [123]. These findings, in agreement with previous studies on buck [124] and other species [125], might indicate that the positive effect of resveratrol on thawed semen could be dependent on dose, sperm variables such as concentration used for freezing, animal species, storage procedure, and entity of stressing conditions. As discussed until now, the use of resveratrol in sperm preservation has been extensively related to its supplementation to extenders before cryopreservation. However, in a recent study, it was shown that resveratrol supplementation in washing and fertilization media improved fertilization capability of bovine sex-sorted spermatozoa with respect to not-treated ones, increasing blastocyst percentage and quality following IVF [126]. This occurred because the spermatozoa had a decreased oxidative stress, since mitochondrial function and acrosomal integrity were ameliorated [126]. The results of this study open the stage for new applications related to the use of resveratrol in the field of artificial reproductive techniques.

**Figure 3:**
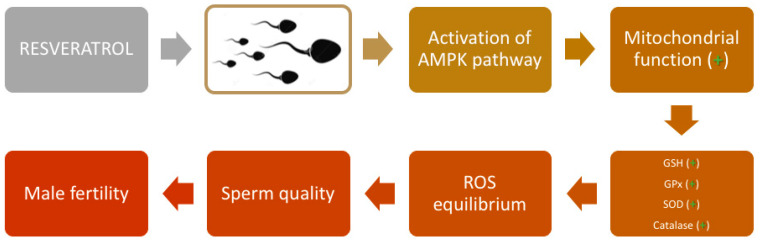
Mechanism of action of resveratrol in spermatozoa. Resveratrol activates the AMPK pathway, which is correlated with a higher mitochondrial function and higher activity of Glutathione (GSH), glutathione peroxidase (GPx), Superoxide dismutase (SOD), and catalase determining ROS equilibrium. These mechanisms protect against oxidative stress, ameliorating sperm quality and, thereby, fertility of the spermatozoa.

Despite the positive effect of resveratrol in protecting spermatozoa from oxidative stress, the mechanisms of action are still an object of debate. In several studies, it was shown that resveratrol activates the AMPK pathway in spermatozoa (Figure 3).

AMPK is a key kinase involved in regulating the cellular redox state by switching the metabolic pathway under stressful conditions [64,65]. It was observed that resveratrol activated AMPK in somatic cells in vitro [127,128,129]. In human spermatozoa, it was demonstrated that AMP-activated protein kinases are mainly present in the whole flagellum and the post-equatorial region of the head [130]. Related to these findings, supplementation of resveratrol increased AMPK activity and was beneficial for protection against cryopreservation-induced oxidative stress of human spermatozoa by improving DNA integrity and transcripts, which were used as markers of sperm quality [130]. Similar results were obtained in boar [131] and goat [132] spermatozoa. In both studies, the addition of resveratrol activated AMPK phosphorylation, allowing the reduction of ROS production, while enhancing the sperm antioxidative defense system such as GSH level and activities of glutathione peroxidase (GPx), SOD, and catalase (Figure 3). However, while it is well-established that resveratrol is capable of activating AMPK, the exact mechanism by which this occurs remains to be clarified [133]. This is because the activation of AMPK can take place through a variety of complex and apparently contradictory mechanisms, which include an increase in the AMP/ATP ratio [130]; inhibition of mitochondrial ATP synthase [134,135]; ROS (independent of the AMP/ATP ratio) [136,137]; as well as upstream serine/threonine kinases, such as LKB1 (Peutz–Jeghers protein) [138,139] and calcium/calmodulin-dependent protein kinase kinase b (CaMKKb) [140,141].

## 4. Conclusions

Resveratrol is a natural polyphenol with antioxidant, anti-inflammatory, and anti-aging properties. In several studies, it has been shown that resveratrol modulates both female and male reproduction.

In females, resveratrol is considered a phytoestrogen with a chemical structure similar to that of some estrogens. Interestingly, resveratrol is potentially usable alone or in combination with other hormones for its moderate estrogenic effect. Moreover, resveratrol exerts a steroidogenesis modulation in the ovary via sirtuins, especially SIRT1. Finally, resveratrol is a quality enhancer of aged oocytes and a gametes cryo-protectant, with mainly antioxidant and anti-apoptotic effects.

In males, resveratrol modulates the reproductive function by: (1) enhancing the production of testosterone, (2) triggering penile erection, and (3) improving spermatogenesis including sperm differentiation and number in the testes and ejaculate, respectively. The mechanisms of action seem to be exerted by activating the AMPK pathway. Finally, resveratrol is a suitable antioxidant to supplement to semen extenders thanks to its beneficial effect in preserving sperm quality.

However, although considerable research supports the positive impact of resveratrol on human and animal reproduction, further studies are necessary to consolidate the knowledge on the properties of resveratrol and its role in the reproductive functions.
